# Photoluminescence as a Valuable Tool in the Optical Characterization of Acetaminophen and the Monitoring of Its Photodegradation Reactions

**DOI:** 10.3390/molecules25194571

**Published:** 2020-10-07

**Authors:** Monica Daescu, Adelina Matea, Catalin Negrila, Constantin Serbschi, Alina C. Ion, Mihaela Baibarac

**Affiliations:** 1 Laboratory of Optical Processes in Nanostructured Materials, National Institute of Materials Physics, Atomistilor Street 405A, P.O. Box MG-7, 077125 Bucharest, Romania; monica.daescu@infim.ro (M.D.); adelina.udrescu@infim.ro (A.M.); 2 Faculty of Applied Chemistry and Materials Science, University Politehnica of Bucharest, Gh. Polizu St 1-7, 011061 Bucharest, Romania; ac_ion@yahoo.com; 3 Nanoscale Condensed Matter Laboratory, National Institute of Materials Physics, Atomistilor Street 405A, P.O. Box MG-7, 077125 Bucharest, Romania; catalin.negrila@infim.ro; 4 Bioelectronic SRL, Cercelus Street, no.54, Ploiesti, Romania; bioelectronic90@yahoo.com

**Keywords:** acetaminophen, photodegradation, UV light

## Abstract

In this work, new evidence for the photodegradation reactions of acetaminophen (AC) is reported by photoluminescence (PL), Raman scattering and FTIR spectroscopy. Under excitation wavelength of 320 nm, AC shows a PL band in the spectral range of 340–550 nm, whose intensity decreases by exposure to UV light. The chemical interaction of AC with the NaOH solutions, having the concentration ranging between 0.001 and 0.3 M, induces a gradual enhancement of the photoluminescence excitation (PLE) and PL spectra, when the exposure time of samples at the UV light increases until 140 min, as a result of the formation of p-aminophenol and sodium acetate. This behavior is not influenced by the excipients or other active compounds in pharmaceutical products as demonstrated by PLE and PL studies. Experimental arguments for the obtaining of p-aminophenol and sodium acetate, when AC has interacted with NaOH, are shown by Raman scattering and FTIR spectroscopy.

## 1. Introduction

Acetaminophen (AC) is an active compound of different drugs marketed under the name of Paracetamol, Theraflu, Coldrex, Parasinus, Algopirin, and so on. The therapeutic effects for which AC is administered are: (i) the reduction of headaches during migraines [1]; (ii) the diminution of kidney stone-induced pain [2]; (iii) osteoarthritis which involves the pain at the hip, knee and hands [3]; (iv) the cutting down of the pain resulting after dental procedures [4]; (v) fighting fever [5] and recently in coronavirus disease (Covid-19) [6]. The most important adverse effects of AC reported until now consist of the generation of liver damage [7,8]. The use of a dose of AC higher than 4000 mg per day induces the appearance of acute liver failure, which then leads to the death of the un-treated patients [9].

For the quantitative determination of paracetamol in biological fluids and tablets, a sustained effort was carried out in the development of analytical techniques such as high-performance liquid chromatography (HPLC) [10], UV-VIS absorption spectroscopy [11], surface enhanced Raman scattering [12], cyclic voltammetry [13], square-wave adsorptive anodic stripping voltammetry [14], chronoamperometry [15], differences pulse voltammetry [16], and so on. The drug stability is an important topic with consequences for its therapeutic effect. The tests of the drug stability include the reactions of these with acids and alkaline solution. The AC degradation was studied for the first time in 2013 [17], by the UV-VIS absorption spectroscopy studies, which were validated by HPLC. According to Feng et al. (2013) [17], the alkaline degradation of AC leads to the formation of p-aminophenol, a compound with high toxicity, which was highlighted by UV-VIS spectroscopy over 24 h. In contrast with this progress, we demonstrated that an alternative method to assess the AC degradation in the presence of alkaline solution may be photoluminescence (PL). Both PL and photoluminescence excitation (PLE) are two methods which will allow highlighting the degradation of AC in sodium hydroxide (NaOH) in a shorter time than that reported by UV-VIS absorption spectroscopy. In addition, new evidence for the generation of p-aminophenol and sodium acetate as a result of the interaction of AC with NaOH will be shown by Raman scattering and FTIR spectroscopy.

## 2. Materials and Methods

### 2.1. Materials

The chemical compounds, namely AC and NaOH, were purchased from Sigma–Aldrich. The drugs containing AC as an active compound, marked under the name Paracetamol (containing 500 mg AC, Magistra C&C), Parasinus (having 500 mg AC, Europharm) and Pararemin (having 250 mg AC, Polisano), were bought from a local pharmacy. The composition of a tablet of: (i) Paracetamol from Magistra C&C contains 500 mg AC and excipients of the type povidone, corn starch, croscarmellose sodium and stearic acid; (ii) Parasinus contains as active compounds 500 mg AC, 3 mg chlorpheniramine maleate, 30 mg pseudoephedrine hydrochloride and excipients of the type pregelatinized starch, talc, povidone, stearic acid, magnesium stearate; and (iii) Pararemin contains as active substances 250 mg AC, 150 mg propyphenazone, 50 mg caffeine and excipients of the type pregelatinized starch (whole), pregelatinized starch (partially), polyvinylpyrrolidone, croscarmellose sodium, stearic acid, isomalt, talc, anhydrous colloidal silicon dioxide.

### 2.2. Samples Preparation

To study the behavior of AC in the absence of excipients, an aqueous solution of AC 0.33 M was prepared. The influence of the alkaline solutions on AC was analyzed using aqueous solutions of NaOH with various concentrations. Thus, in all experiments, 2 mL of AC 0.33 M interacted with 1 mL NaOH 10^−3^, 10^−2^, 10^−1^ or 0.3 M.

A tablet of each drug was dispersed into 10 mL distilled water, under ultrasonication, for 15 min. In order to obtain a clear solution, a successively filtration was carried out. For each drug containing AC, i.e., Paracetamol, Parasinus and Pararemin, 2 mL of pharmaceutical compounds interacted with 1 mL NaOH 0.3 M.

The solid-state interaction of AC with NaOH, at a non-hydrostatic pressure equal to 0.58 GPa for 5 min has involved: (a) the preparation of three mixtures of AC and NaOH as follows: (i) 0.168 g AC and 0.04 g NaOH; (ii) 0.1 g AC and 0.2 g NaOH; and (iii) 0.16 g AC and 0.4 g NaOH. The weight ratios of the three mixtures of AC and NaOH are equal to 4.2, 0.5 and 0.4. Homogenization of each mixture of AC and NaOH was performed by grinding for 1 min.

### 2.3. Photodegradation of Samples

The samples were exposed successively at UV light, for 140 min, using a mercury-vapors lamp with the power of 350 W, by the selecting with an UG5 filter of the spectral range 200–380 nm, that contains the Hg spectrum line of high intensity at 253 nm.

### 2.4. Samples Characterization

PL and PLE spectra of the AC aqueous solution in the absence and in the presence of NaOH were recorded, in the right-angle geometry, with a Fluorolog-3 spectrophotometer, FL3-2.2.1 model, from Horiba Jobin Yvon, endowed with a 450 W Xe lamp.

Raman spectra of AC before and after the interaction with NaOH in the solid state were recorded with a FT Raman spectrophotometer, RFS100S model, from Bruker, endowed with a YAG:Nd laser (excitation wavelength 1064 nm).

IR spectra of AC in the initial state and after the interaction with NaOH in the solid state were recorded with an FTIR spectrophotometer, Vertex 80 model, from Bruker, in the attenuated total reflection geometry.

Additional information concerning the products resulting from the interaction of AC with NaOH are shown by X-ray photoelectron spectroscopy (XPS), using a SPECS spectrometer endowed with a PHOBIOS 150 analyzer and an Al Kα source.

## 3. Results

**Figure 1:**
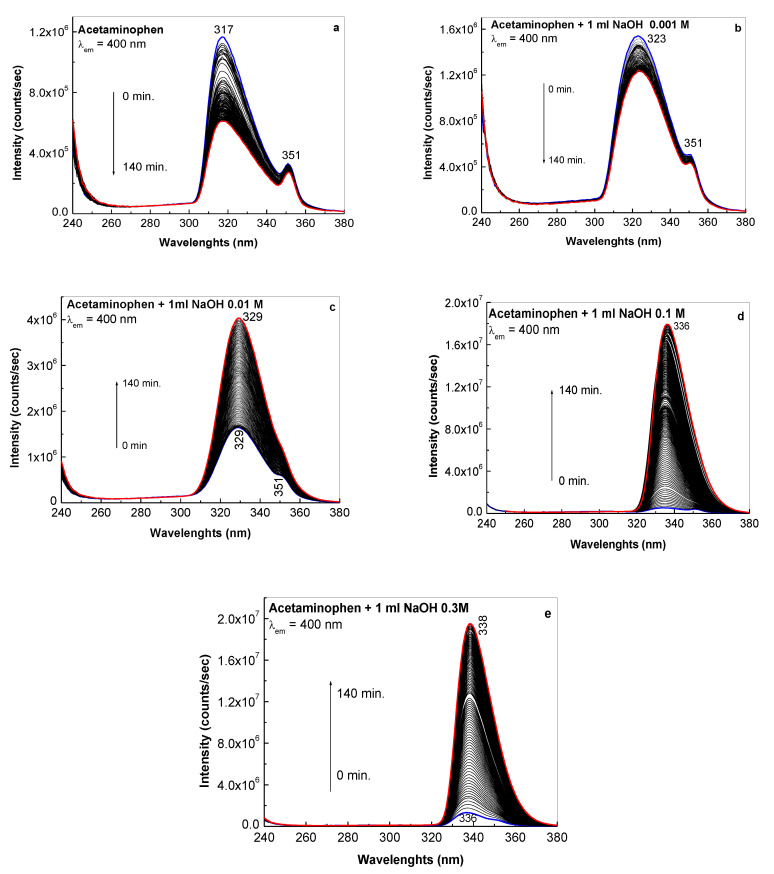
The photoluminescence excitation (PLE) spectra of the aqueous solution of acetaminophen (AC) before (**a**) and after the interaction with the aqueous solution of NaOH 10^−3^ M (**b**), 10^−2^ M (**c**), 10^−1^ M (**d**) and 3 x 10^−1^ M (**e**). All PLE spectra were recorded at the emission wavelength of 400 nm. Blue and red curves correspond to the first and last spectrum, respectively, of the samples exposed to UV light for 0 and 140 min. Black curves correspond to the intermediate spectra of above samples, each PLE spectrum being collected at 84 s, respectively.

According to Figure 1a, the PLE spectrum of the AC aqueous solution shows an intense band with the maximum at 317 nm, which is accompanied of another of low intensity at 351 nm. An important decrease in the intensity of the band at 317 nm is observed to occur from 1.17 × 10^6^ to 6.1 × 10^5^ counts/sec when AC is exposed to UV light for 140 min (Figure 1a). The interaction of AC with the NaOH solutions having concentrations 10^−3^, 10^−2^, 10^−1^ and 0.3 M induces an up-shift of the PLE band of AC from 317 nm (Figure 1a) to 323 nm (Figure 1b), 329 nm (Figure 1c), 336 nm (Figure 1d) and 338 nm (Figure 1e), respectively.

**Scheme 1:**
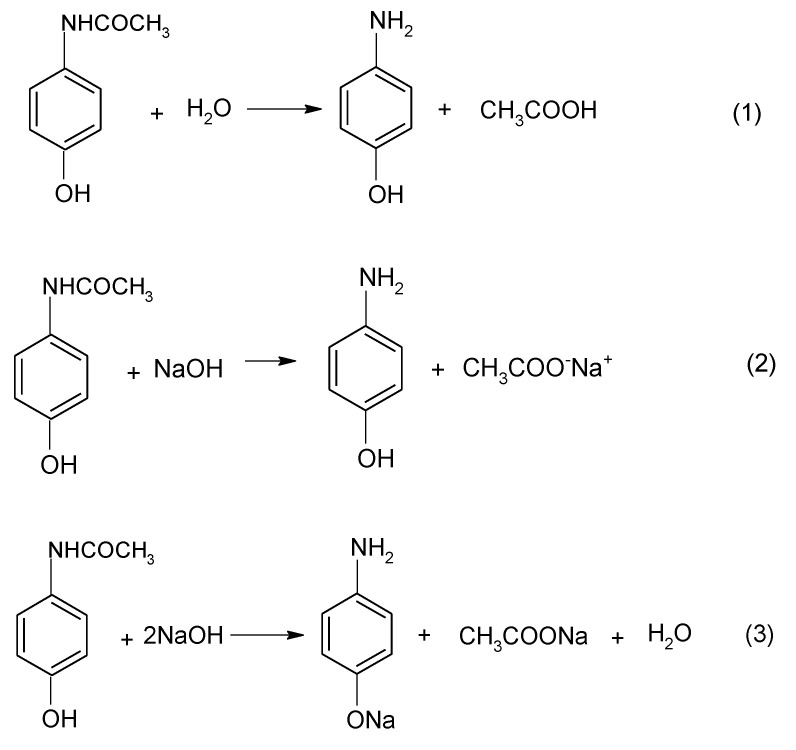
The chemical reactions of AC with H_2_O (Equation (**1**)) and NaOH (Equation (**2**)). The interaction of AC with an excess of NaOH is described by Equation (**3**).

**Figure 2:**
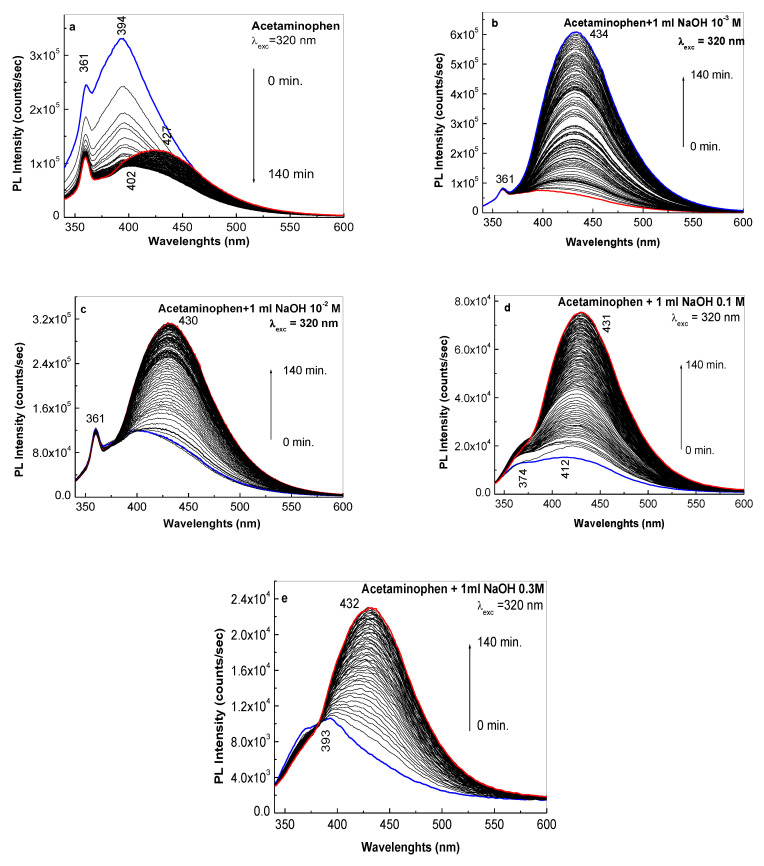
The photoluminescence (PL) spectra of the aqueous solution of AC before (**a**) and after the interaction with the aqueous solution of NaOH 10^−3^ (**b**), 10^−2^ (**c**), 10^−1^ (**d**) and 3 × 10^−1^ M (**e**). All PL spectra were recorded at the excitation wavelength of 320 nm. Blue and red curves correspond to the first and last spectrum, respectively, of the samples exposed to UV light for 0 and 140 min. Black curves correspond to the intermediate spectra of above samples, each PL spectrum being collected at 140 s, respectively.

Figure 2 highlights changes in the PL band position and intensity of AC when an interaction of this with the NaOH solutions having concentrations 10^−3^, 10^−2^, 10^−1^ and 0.3 M occurs. The PL spectrum of AC shows two emission bands at 361 and 394 nm, the last one having the intensity equal to 3.3 × 10^5^ counts/sec (Figure 2a). As observed in Figure 2a, the exposure of the AC aqueous solution to UV light, for 140 min, leads to a shift of the maximum of emission band from 394 to 427 nm simultaneously with a decrease in the intensity of this band from 3.3 × 10^5^ to 1.24 × 10^5^ counts/sec. The interaction of the AC with NaOH solutions having concentrations 10^−3^, 10^−2^, 10^−1^ and 0.3 M induces a variation in the intensity of the PL spectrum at cca. 7.3 × 10^4^, 1.18 × 10^5^, 1.5 × 10^4^ and 1 × 10^4^ counts/sec, respectively. This result can be explained considering the decrease in AC concentration in the reaction mixture as a consequence of the chemical interaction of AC with NaOH, according to reaction (2) in Scheme 1. Regardless of the concentration of the NaOH solution that interacted with the AC, after 140 min of exposure to UV light, it is noticed that the PL spectra shown in Figure 2b-e have maxima at approx. 430–434 nm. According to Figure 2b–e, after 140 min of exposure to UV light, the AC interacted with the NaOH solutions having the concentrations equal to 10^−3^, 10^−2^, 10^−1^ and 0.3 M, the intensity of the PL spectra is equal to 6.09 × 10^5^, 3.1 × 10^5^, 7.5 × 10^4^ and 2.3 × 10^4^ counts/sec, respectively.

A similar behavior with that reported in the case of AC (Figure 1a) takes place by the interaction of AC with NaOH 10^−3^ M (Figure 1b). In this last case, one observes for the PLE band at 323 nm, a decrease in intensity from 1.54 × 10^6^ to 1.22 × 10^6^ counts/sec. For higher NaOH concentrations such as those in the 0.01–0.3 M range, an inverse behavior is reported by exposure to UV light of AC solutions which interact with NaOH. By increasing the exposure time at UV light up to 140 min, according to Figure 1c–e, an intensity increase of the PLE band of AC interacted with 10^−2^, 10^−1^ and 0.3 M NaOH solutions from 1.64 × 10^6^, 5.18 × 10^5^ and 1.32 × 10^6^ counts/sec to 4.03 × 10^6^, 1.79 × 10^7^ and 1.94 × 10^7^ counts/sec, respectively, takes place.

**Figure 3:**
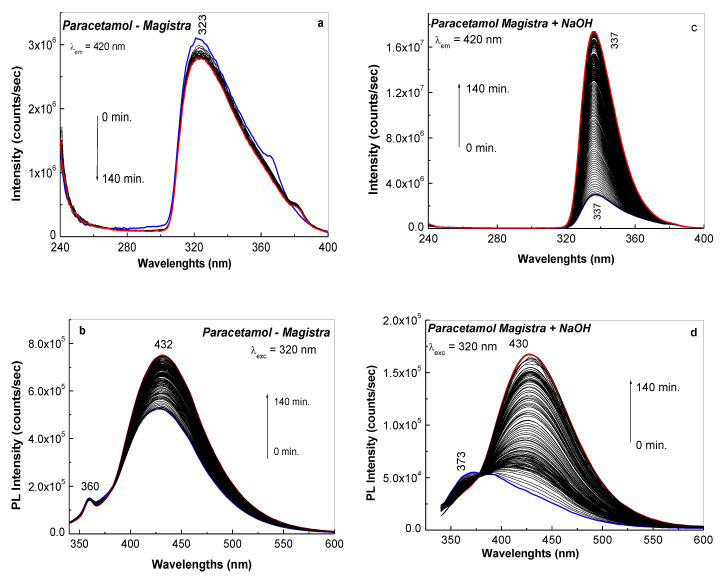
The PLE and PL spectra of the aqueous solution of Paracetamol before (**a**,**b**) and after the interaction with the aqueous solution of NaOH 3 × 10^−1^ M (**c**,**d**). Blue and red curves correspond to the first and last spectrum, respectively, of the samples exposed to UV light for 0 and 140 min. Black curves correspond to the intermediate spectra of above samples, each PLE and PL spectrum being collected at 84 and 140 s, respectively.

**Figure 4:**
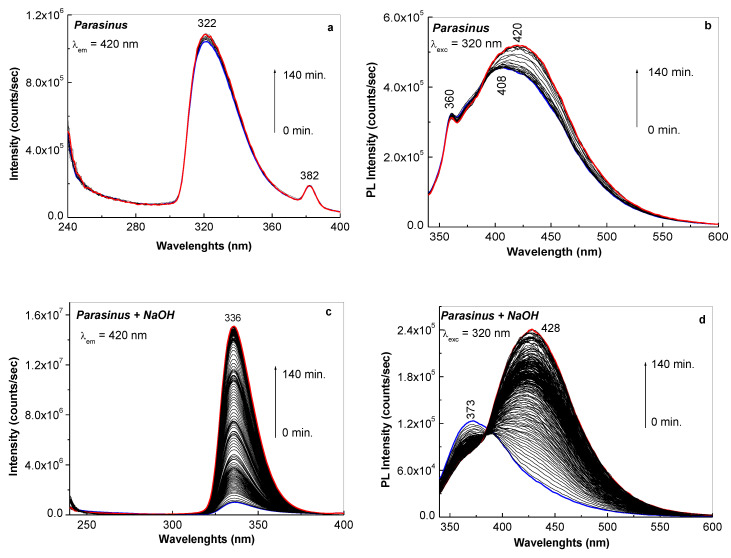
The PLE and PL spectra of the aqueous solution of Parasinus before (**a**,**b**) and after the interaction with the aqueous solution of NaOH 3 × 10^−1^ M (**c**,**d**). Blue and red curves correspond to the first and last spectrum, respectively, of the samples exposed at UV light for 0 and 140 min. Black curves correspond to the intermediate spectra of above samples, each PLE and PL spectrum being collected at 84 and 140 s, respectively.

**Figure 5:**
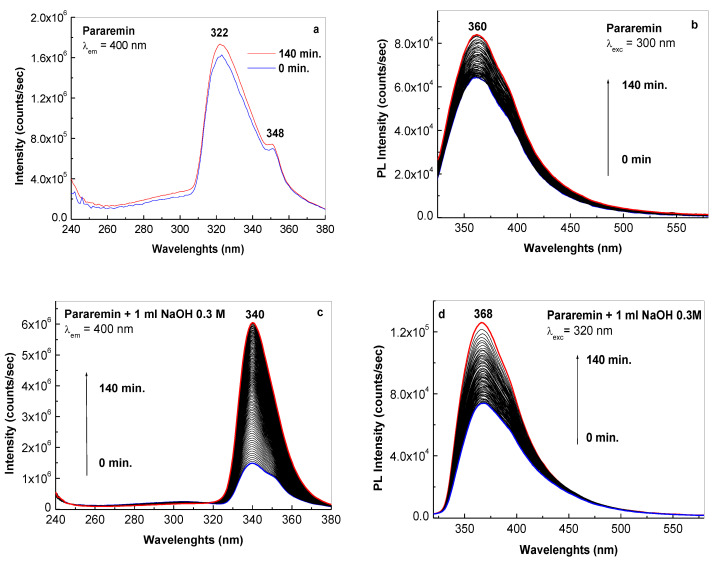
The PLE and PL spectra of the aqueous solution of Pararemin before (**a**,**b**) and after the interaction with the aqueous solution of NaOH 3 × 10^−1^ M (**c**,**d**). Blue and red curves correspond to the first and last spectrum, respectively, of the samples exposed to UV light for 0 and 140 min. Black curves correspond to the intermediate spectra of above samples, each PLE and PL spectrum being collected at 84 and 140 s, respectively.

These variations in the PLE and PL spectra of AC are also found in the case of pharmaceutical compounds, after the filtering process. In order to support this statement, Figure 3, Figure 4 and Figure 5 show the PLE and PL spectra of the pharmaceutical compounds marketed under the name of Paracetamol, Parasinus and Pararemin before and after their interaction with aqueous solution of NaOH 0.3 M.

In the presence of excipients, the band of the AC PLE spectrum is shifted to 323 nm. By exposure to UV light of the aqueous solution of Paracetamol, containing AC and excipients, for 140 min, a variation in the intensity of the PLE band is observed from 3.1 × 10^6^ to 2.7 × 10^6^ counts/sec (Figure 3a). The interaction of the aqueous solution of Paracetamol with NaOH leads to a shift of the PLE band from 323 nm (Figure 3a) to 337 nm (Figure 3c), the subsequent exposure at UV light for 140 min inducing an intensity increase of the PLE band from 2.98 × 10^6^ to 1.74 × 10^7^ counts/sec. Regarding the PL spectrum of Paracetamol, it is observed that the most intense emission band from 394 nm (Figure 3a) is shifted at 434 nm (Figure 3b), while the lowest intensity band has the maximum at 361 nm (Figure 3b), i.e., at the same wavelength as the PL spectrum of AC in the absence of excipients (Figure 3a). By exposure to UV light of the aqueous solution of Paracetamol, for 140 min, a variation of the intensity of the PL band is noticed from 5.22 × 10^5^ to 7.49 × 10^5^ counts/sec. The interaction of Paracetamol with NaOH induces a down-shift of emission band from 360 nm (Figure 3b) to 373 nm (Figure 3d) and subsequent exposure to UV light induces a gradual up-shift of the band from 373 at 430 nm simultaneous with an intensity increase of this emission band from 5.38 × 10^4^ to 1.7 × 10^5^ counts/sec (Figure 3d). These results confirm that the excipients do not influence the evolution of PLE and PL spectra under UV light.

Table 1 shows the variations induced in the PL and PLE spectra of AC in the presence of other active compounds and excipients, for the particular cases of Parasinus and Pararemin, the results being shown in Figure 4 and Figure 5.

The increase in the intensity of the PLE and PL spectra of AC in the presence of other active compounds and excipients (Figure 4; Figure 5) is similar to that reported in the case of pure active compounds (Figure 1; Figure 2). To quantify the impact of additional active compounds on AC in the particular case of Parasinus and Pararemin, a careful analysis of Figure 4 and Figure 5 is necessary. In this context, one remarks that: (i) in the initial state, for the two pharmaceutical formulations the maximum of the PLE band is situated at 322 nm, the variation in the intensity of the PLE band, when the samples were exposed to UV light for 140 min, being only of 4.5% and 7.6% in the case of Parasinus (Figure 4a) and Pararemin (Figure 5a), respectively. These values are lower than those reported in the case of AC and Paracetamol, when one remarks a variation in the intensity of the PLE bands with maxima at 317 and 323 nm of 47.8% (Figure 1a) and 11.9% (Figure 3a). Changes in the intensities of the PL bands of Parasinus and Pararemin, with maxima located at 420 and 360 nm are reported to be equal to 12.8% (Figure 4b) and 23.8% (Figure 5b), respectively. These variations are smaller compared to those of AC and Paracetamol, for which variations of ~63.6% and ~30.3% in intensity of the PL bands with maxima at 394 nm (Figure 2a) and 432 nm (Figura 3b), respectively, are reported; ii) after 140 min of exposure to UV light of the two pharmaceutical products, which were interacted with 1 mL NaOH 0.3M, an increase in intensity of PLE bands of Parasinus and Pararemin of ~93.2% (Figure 4c) and 75.5% (Figure 5c) was reported. These values demonstrate that the presence of chlorpheniramine maleate and pseudoephedrine hydrochloride does not induce additional changes in comparison with those of AC interacting with NaOH, and by successive exposure at UV light for 140 min, an increase in the intensity of the PLE band of 93.2% (Figure 1e) was noticed. Referring to AC (Figure 1e), the presence of propyphenazone and caffeine induces a slight inhibition of the photodegradation process of AC according to Figure 5c. As increasing the weight of the additional active compounds to AC from 0.6 and 44 wt.%, a reduced variation of the intensity of PL spectra of Paracetamol, Parasinus and Pararemin, from 68% to 49.8% and 42% respectively, is reported. This result confirms that the active compounds contained in Parasinus (chlorpheniramine maleate and pseudoephedrine hydrochloride) and Pararemin, (propyphenazone and caffeine) have the role to inhibit the photodegradation of AC in the alkaline environment.

These changes reported in Figure 1, Figure 2, Figure 3, Figure 4 and Figure 5 can be explained if we take into account the chemical reactions shown in Scheme 1. According to Scheme 1, depending on the molar ratio between AC and NaOH, i.e., 1:1 (Equation (2)) or 1:2 (Equation (3)), the reaction products correspond to sodium acetate and p-aminophenol or sodium p-aminophenoxide, respectively.

**Figure 6:**
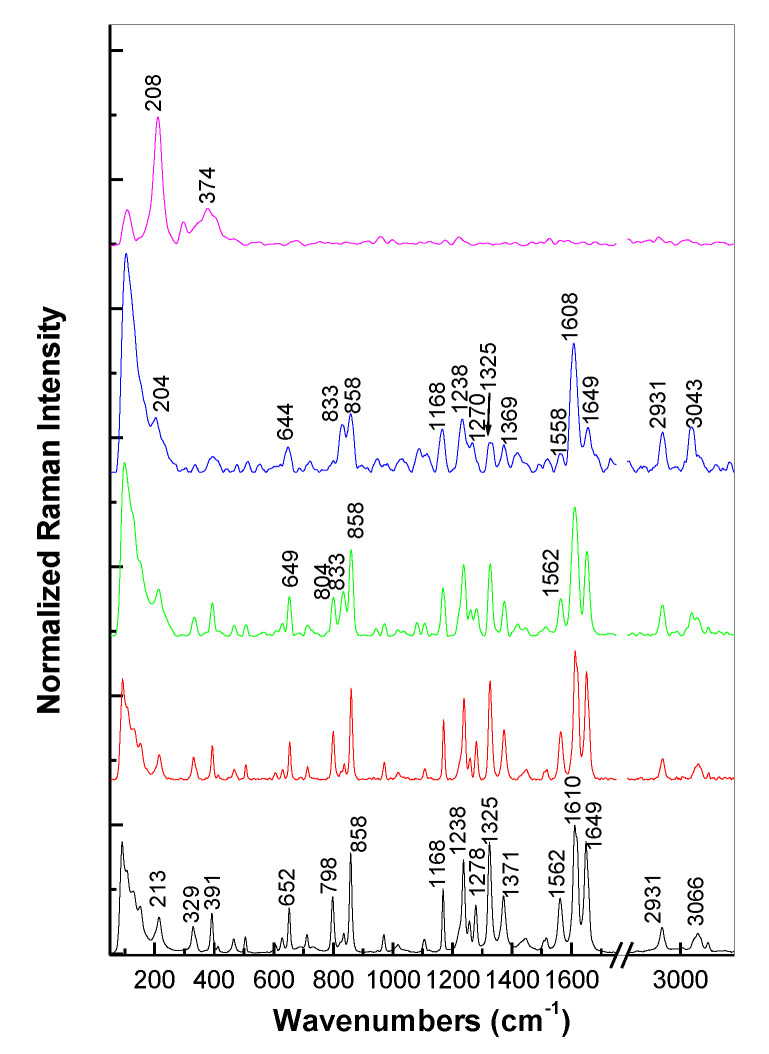
The Raman spectrum of AC (black curve). Red, green and blue curves correspond to the Raman spectra of AC interacted with NaOH, and subsequently exposed to UV light for 140 min, when the AC: NaOH weight ratio is equal to 4.2, 0.5 and 0.4, respectively. Magenta curve corresponds to the Raman spectrum of NaOH.

**Figure 7:**
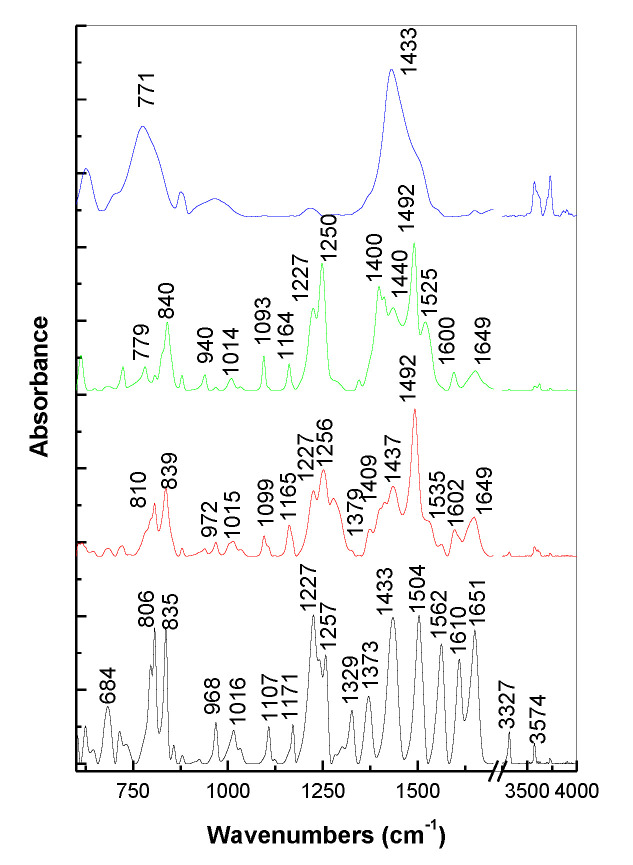
The IR spectra of AC before (**black** curve) and after the interaction with NaOH, when the AC: NaOH weight ratio is equal to 4.2 (**red** curve) and 0.4 (**green** curve), the samples being subsequently exposed to UV light for 140 min. **Blue** curve corresponds to the IR spectrum of NaOH.

Figure 6 and Figure 7 prove that the chemical reactions of AC with NaOH, shown in Scheme 1, lead to the generation of p-aminophenol and sodium acetate. As shown in Table 2, the main drawback of the IR spectroscopy and Raman scattering in comparison with PL is that these methods do not allow acquiring information in dilute analytes solutions such as those used in the PL studies reported above. Therefore, in the future experiments solid state samples were used to illustrate the interaction of AC with NaOH.

In Figure 6, the black curve shows the Raman spectrum of AC, which is characterized by the lines peaked at 391, 652, 798, 858, 1168, 1238, 1278, 1325, 1562, 1610, 1649, 2931 and 3066 cm^−1^, that are attributed to the vibrational modes of the deformation of phenyl ring + C-H twisting, deformation of phenyl ring, deformation of phenyl ring + C-C rocking in amide group, breathing of phenyl ring, C-O-H bending + C-C-H bending in aryl group, C-N stretching + C-C stretching in amide group, C-O-H bending + C-C-H bending in aryl group + C-N rocking in amide group, C-O-H bending + C-H rocking + C-C stretching in aryl group, C-N-H bending in amide group, C-N stretching + C-O stretching +C-C stretching in aryl group, C=O stretching in amide group, symmetrical C-H stretching in amide group and asymmetrical C-H stretching in amide group, respectively. [18] The interaction of AC with NaOH leads to the variations in Raman spectrum of AC shown in Figure 6 as follows: (i) an up-shift or the Raman line assigned to the vibrational mode of breathing of the phenyl ring from 798 to 804 cm^−1^ simultaneous with its increase in the intensity; (ii) a gradual decrease in the intensity of the Raman lines situated in the spectral range 1100–1400 cm^−1^ as well as the Raman lines assigned to the vibrational modes of C-N-H bending in the amide group and C=O stretching in the amide group peaked at 1562 and 1649 cm^−1^, respectively; (iii) a down-shift of the Raman line from 3066 to 3043 cm^−1^; (iv) a gradual increase in the intensity of the Raman line, assigned to the vibrational mode of C-N stretching + C-O stretching + C-C stretching in aryl group, which is peaked at 1608 cm^−1^; and v) a change of the ratio between the intensities of the Raman lines peaked at 1649 and 2931 cm^−1^ from 4.35 (value reported in the case of AC) to 2.69 and 1.12, when samples contain the AC: NaOH mass ratios equal to 0.5 and 0.4, respectively. A puzzling fact is that the aniline-derived compounds and carboxylic acids show in the Raman spectra, lines at 1605 [19,20] and 2933 cm^−1^ [21], respectively, very close from those reported in Figure 6. Taking into account these facts, in our opinion, the increase in the relative intensity of the Raman lines at 1608 and 2931 cm^−1^ originates in the formation of p-aminophenol and sodium acetate, respectively.

The IR spectrum represented by black curve in Figure 7 shows IR bands of AC, peaked at 684, 806, 835, 968, 1016, 1107, 1171, 1227, 1257, 1329, 1373, 1433, 1504, 1562, 1610, 1651, 3327 and 3574 cm^−1^, belonging to the vibrational modes of: the phenyl deformation + C-C stretching in amide group, phenyl ring deformation + C-C rocking in amide group, C-C-H bending in aryl group, N-C=O bending in amide group, C-C-H bending in amide group, C-C rocking + C-O-H bending in aryl group, C-O-H bending + C-C-H bending in aryl group, C-N stretching + C-C stretching in amide group, C-N-H bending in amide group + C-O stretching + C-O-H bending in aryl group, C-O-H bending + C-H rocking + C-C stretching in aryl group, C-H rocking in amide group, C-O-H bending in aryl group + C-N-H bending in amide group, C-H rocking in amide group, C-N-H bending in amide group, C-N stretching + C-O stretching + C-C stretching in aryl group, C=O stretching in amide group, N-H stretching in amide group and O-H stretching, respectively. [18] As increasing NaOH weight that interacts with AC, the following variations are induced in Figure 7: (i) an up-shift of the IR band assigned to the vibrational mode C-C-H bending in aryl group from 835 to 840 cm^−1^, simultaneous with the decrease of the absorbance of IR band attributed to the phenyl ring deformation + C-C rocking in amide group vibrational mode; (ii) the decrease in the absorbance of the IR bands localized in the spectral ranges 900–1200 and 1373–1379 cm^−1^ and the down-shift of the IR bands from 968, 1107 and 1171 cm^−1^ to 940, 1093, 1164 cm^−1^, respectively; (iii) the increase in the absorbance of the IR bands situated in the spectral range 1400–1500 cm^−1^ and the down-shift of the IR bands from 1504 and 1562 cm^−1^ to 1492 and 1525 cm^−1^; and (iv) the gradual decrease in the absorbance of the IR bands localized in the spectral ranges 1590–1660 and 3300–3700 cm^−1^. The absence of the IR bands of 1329, 1373 and 3327 cm^−1^ as well as the diminution of the absorbance of IR bands localized in the spectral ranges 1510–1700 and 3500–3600 cm^−1^ simultaneous with the increase of the absorbance of IR band at 1492 cm^−1^ attributed to the C=C stretching vibrational mode in compounds of the type aniline [20,22] indicate a decrease in the amide group weight as a consequence of the appearance of the amine and carboxylic groups, as shown in Scheme 1. A decrease in the amide group weight after the interaction of AC with NaOH is also observed by XPS spectroscopy.

**Figure 8:**
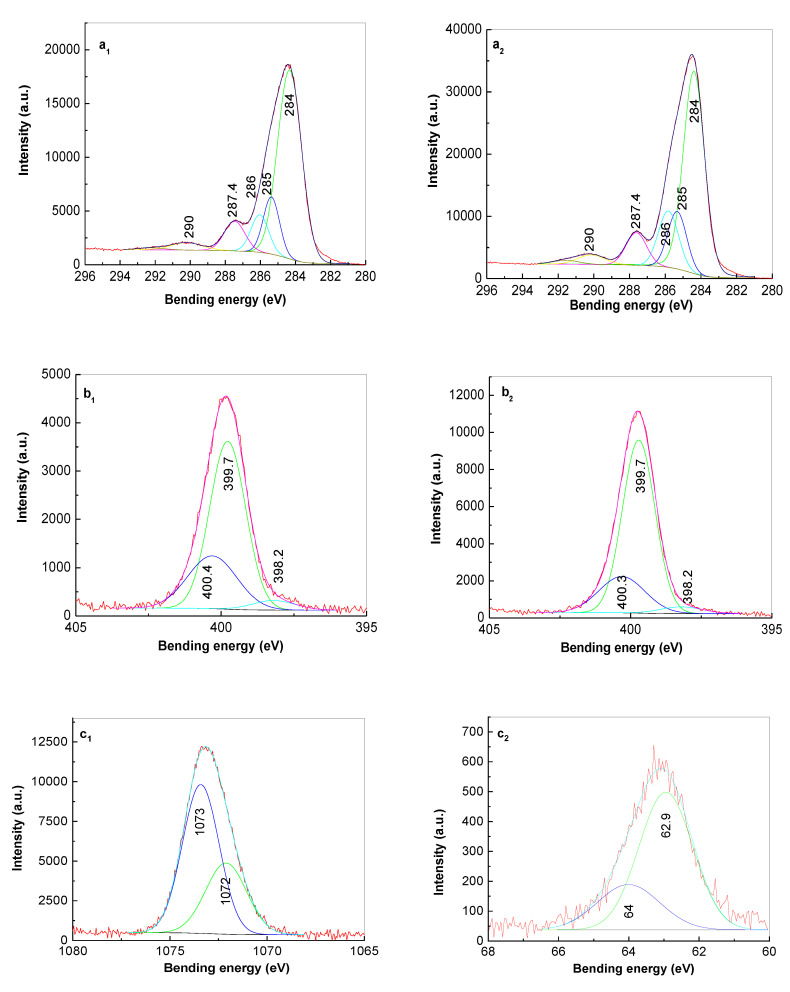
XPS C1s (**a**) and N1s (**b**) spectra of AC (**a_1_**,**b_1_**) as well as AC interacted with NaOH (**a_2_**,**b_2_**). (**c_1_**,**c_2_**) show XPS Na1s and Na2s spectra of AC interacted with NaOH.

According to Figure 8a_1_, the XPS C1s spectrum of AC is characterized by the five peaks at 284, 285, 286, 287.4 and 290 eV, assigned to the following bonds: C=C, C-N, C-O in phenolic compounds, C=O in amide and C=O/C=C in aromatic ring, respectively. [23] Two peaks are observed in the N1s spectrum of AC (Figure 8b_1_) at 400.4 and 399.7 eV which were attributed to the –NHCO and –NH– bonds. [23] In contrast with AC, the following variations are highlighted in Figure 8, as a consequence of the interaction of AC with NaOH: (i) an increase of the intensity of the peak at 284 eV; (ii) a decrease of the intensity ratio of the peaks at 400.4 and 399.7 eV from 0.34 to 0.23 (Figure 8b_1_,b_2_), and iii) the presence of the Na2s peaks at 62.9 and 64 eV in Figure 8c_2_, which were attributed to CH_3_COONa and un-reacted NaOH [24,25], respectively. The Na1s spectrum was also fitted with two components (Figure 8c_1_)—the peak at 1073 eV attributed to NaOH and another one at 1072 eV attributed to CH_3_COONa. Due to the large difference in kinetic energy of the photoelectrons from Na2s and Na1s orbitals, it is proper to assume that the Na1s photoelectrons describe the first layers of the sample’s surface while the Na2s photoelectrons describe the deeper layers. Comparing the peak intensities in the two spectra we can deduce that the unreacted NaOH is only on the surface of the sample (Figure 8c_1_,c_2_). All these changes plead for the formation of p-aminophenol and sodium acetate. Based on these results, the most important advantages and drawbacks of the PL in comparison with other methods used in the assessing of the photodegradation processes of the pharmaceutical compounds are shown in Table 2.

## 4. Conclusions

Using photoluminescence (PL), Raman scattering and FTIR spectroscopy, new evidence concerning the interaction of AC with NaOH was reported in this work. Our results lead to the following conclusions: (i) photoluminescence represents an alternative method to UV-VIS spectroscopy to monitor the reaction of AC with NaOH under UV light; this reaction is highlighted by the up-shift of the PLE and PL bands from 317 and 394 nm to 338 and 432 nm, respectively, simultaneous with an intensity increase of these spectra; (ii) the PLE and PL studies have demonstrated that this photochemical reaction is not influenced by the excipients existing in pharmaceutical compounds or the other active compounds such as those used in antipyretic drugs; (iii) the changes invoked in the emission spectra are a consequence of the fact that the interaction of AC with NaOH results in the formation of p-aminophenol and sodium acetate; (iv) the main evidence shown in Raman spectra for the formation of p-aminophenol and sodium acetate consist of the presence of the line at 1608 cm^−1^ and the change of the ratio between the relative intensities of the Raman lines at 1649 and 2931 cm^−1^ in the favor of the last one; the Raman lines at 1608 and 2931 cm^−1^ correspond to the C=C stretching vibrational mode in the aniline-derive compound and carboxylic group, respectively; and (v) diminution of the amide group weight in AC as a consequence of the interaction with NaOH was highlighted in IR spectra by the absence of the bands at 1329, 1373 and 3327 cm^−1^, the decrease in the absorbance of the IR bands situated in the spectral ranges 1510–1700 and 3500–3600 cm^−1^ and the presence of the IR band at 1492 cm^−1^ attributed to the C=C stretching vibrational mode in aniline-derive compounds. A diminution of the amide group simultaneous with the formation of the amine and carboxylic groups, as a consequence of the interaction of AC with NaOH, was also highlighted by XPS spectroscopy.

## Figures and Tables

**Table 1:** The dependence of the maximum and the intensity of PLE and PL spectra of Parasinus and Pararemin as depending on the exposure time to UV light and the interaction with NaOH.

Drug Name	Conc. NaOH (M)	Exposure Time at UV Light (min)	λ_PLE_ (nm)	λ_PL_ (nm)	I_PLE_ (counts/sec)	I_PL_ (counts/sec)
Parasinus	0	0	322	408	1.04 × 10^6^	4.55 × 10^5^
		140	322	420	1.09 × 10^6^	5.22 × 10^5^
	0.3	0	336	373	1.02 × 10^6^	1.23 × 10^5^
		140	336	428	1.5 × 10^7^	2.45 × 10^5^
Pararemin	0	0	322	360	1.6 × 10^6^	6.4 × 10^4^
		140	322	360	1.73 × 10^6^	8.4 × 10^4^
	0.3	0	340	368	1.48 × 10^6^	7.36 × 10^4^
		140	340	368	6.05 × 10^6^	1.27 × 10^5^

**Table 2:** The most important advantages and drawbacks of the PL in comparison with other methods to assess the photodegradation processes of the pharmaceutical compounds.

PL Advantages	PL Drawbacks	PL vs. Another Method	References
Shorter time of collecting results in the case of the samples in powder state or as solutions	High cost of infrastructure	UV-VIS spectroscopy	This work; [11]
Shorter time of collecting results; no need additional reagents; the photodegradation process can be carried both in-situ and ex-situ	Unseparated products from the reaction mixture	HPLC	This work; [10]
In PL studies, pharmaceutical products can be both as powder, tablet or dilute solutions; the photodegradation process can be analyzed both in-situ and ex-situ.	High cost of infrastructure; higher time of collecting results; require information about the chemical structure of photo-generated products	IR spectroscopy	This work
Pharmaceutical products can be analyzed in the form of dilute solutions; the photodegradation process can be analyzed both in-situ and ex-situ.	Higher time of collecting results	Raman scattering	This work
Pharmaceutical products can be analyzed in the form of dilute solutions	Require information about the chemical structure of photo-generated products	XPS	This work

## References

[1] Haag G., Diener H.C., May A., Meyer C., Marck H., Straube A., Wessely P., Evers S. (2011). Self-medication of migraine and tension type headache summary of the evidence based recommendations of the Deutsche Migrane und Kopbschmerzgesellschaft (DMKG), the Deutsch Gesellschaft fur Neurologie (DGN), the Osterreichische Kopfschmerzgesellschaft (OKSG) and the Schweizerische Kopfwegesellschaft (SKG). J. Headache Pain.

[2] Saragiotto B.T., Abdel Shaheed C. (2019). Paracetamol for paint in adults. BMJ.

[3] Hochberg M.C., Altman R.D., April K.T., Benkhalti M., Guyatt G., McGowan J., Towheed T., Welch V., Wells G., Tugwell P. (2012). American College of Rheumantology 2012 recommendations for the use of nonpharmacology and pharmacologic therapies in osteoarthritis of the hand, hip and knee. Arhritis Care Res..

[4] Moore R.A., Derry C. (2013). Efficacy of OTC analgesics. Int. J. Clin. Pract. Suppl..

[5] Perrotd D.A., Pira T., Goodenough B., Champion G.D. (2004). Efficacy and safety of acetaminophen vs. ibuprofen for treating children’s pain or fever: A meta-analysis. Arch. Pediatr. Adolesc. Med..

[6] Tan S.H.S., Hong C.C., Saha S., Murphy D., Hui J.H. (2020). Medications on COVID-19 patients: Summarizing the current literature from an orthopedic perspective. Int. Orthop..

[7] Lee W.M. (2017). Acetaminophen (APAP) hepatotoxicity isn’t it time for APAP to go away?. J. Hepatol..

[8] Larson A.M., Polson J., Fontana R.J., Davern T.J., Lalani E., Hynan L.S., Reisch J.S., Schiodt F.V., Ostapowicz G., Shaki A.O. (2005). The acute live failure study group, acetaminophen induced acute liver failure: Results of a United States multicenter, perspective study. Hepatology.

[9] Rumack B.M. (1975). Acetaminophen poisoning and toxicity. Pediatrics.

[10] Bosch M.E., Sauche A.J.R., Rojas F.S., Ojeda C.B. (2006). Determination of paracetamol: Historical evolution. J. Pharm. Biomed. Anal..

[11] Ruiz-Medina A., Fernandez-de Cordona M.L., Ayora-Canada M.J., Pascual-Reguera M.I., Molina-Diaz A. (2000). A flow-through solid phase UV spectrophotometer biparameter sensor for the sequential determination of ascorbic acid and paracetamol. Anal. Chim. Acta.

[12] Santos E.D.B., Lima E.C.N.L., De Oliveira C.S., Sigoli F.A., Mazali I.O. (2014). Fast detection of paracetamol on a fold nanoparticle-chitosan substrate by SERS. Anal. Met..

[13] Norrandri I., Rakhmana R. (2012). Carbon paste electrode modified with poly(3-aminophenaol) for voltammetric determination of paracetamol. Int. J. Electrochem. Sci..

[14] Kutluay A., Aslanoglu M. (2014). An electrochemical sensor prepared by sonochemical one-pot synthesis of multi-walled carbon nanotube-supported cobalt nanoparticles for the simultaneous determination of paracetamol and dopamine. Anal. Chem. Acta.

[15] Adhikari B.R., Govindhen M., Chen A. (2015). Sensitive detection of acetaminophen with graphene based electrochemical sensor. Electrochim. Acta.

[16] Arvand M., Gholizadeh T.M. (2013). Simultaneous voltametric determination of tyrosine and paracetamol using a carbon nanotube-graphene nanosheet nanocomposite modified electrode in human blood serum and pharmaceuticals. Colloids Surf. B.

[17] Feng X., Zhang Q., Cong P., Zhu Z. (2013). Determination of the paracetamol degradation process with online UV spectroscopic and multivariate curve resolution—Alternating least squares methods comparative validation by HPLC. Anal. Methods.

[18] Stasilowicz A., Mizera M., Tykarska E., Lewandowska K.L., Miklaszewski A., Cielecka-Piontek J. (2019). The possibility of using X-ray powder diffraction, infrared and Raman spectroscopy in the study of the identification of structural polymorphs of acetaminophen. Acta Pol. Pharm. Drug Res..

[19] Brouwer A.M., Wilbrandt R. (1996). Vibrational spectra of *N*,*N*-dimethylaniline and its radical cation. An interpretation based on quantum chemical calculations. J. Phys. Chem. C.

[20] Shukla A.R., Asthana B.P., Dongre N.G., Pathak C.M. (1992). Infrared and Raman spectra of non-vicinally trisubstituted 2, 5-dimethylaniline and 2-methyl-5-chloroaniline. Vibr. Spectrosc..

[21] Koleva V., Stoilova D. (2002). Infrared and Raman studies of the solids in the Mg(CH_3_COO)_2_—Zn(CH_3_COO)_2_—H_2_O system. J. Molec. Struct..

[22] Wang S., Li L., Wang Q., Fan Y., Shen J., Zhang K., Yang L., Zhang W. (2018). Aniline oligomer-modified graphene for enhanced electrochemical performances. Synth. Met..

[23] Terzyk A.P. (2001). The influence of activated carbon surface chemical composition of the adsorption of acetaminophen (paracetamol) in vitro Part II. TG, FTIR, and XPS analysis of carbons and the temperature dependence of adsorption kinetics at the neutral pH. Colloid Surf. A Physicochem. Eng. Asp..

[24] Kong X., Castarede D., Boucly A., Artiglia L., Ammann M., Bartels-Rausch T., Thomson E.S., Pettersson J.B.C. (2020). Reversibly physisorbed and chemisorbed water on carboxylic salt surfaces under atmospheric conditions. J. Phys. Chem. C..

[25] Citrin P.H. (1973). High-resolution X-ray photoemission from sodium metal and its hydroxide. Phys. Rev. B.

